# Study of the *cwaRS-ldcA* Operon Coding a Two-Component System and a Putative L,D-Carboxypeptidase in *Lactobacillus paracasei*

**DOI:** 10.3389/fmicb.2020.00156

**Published:** 2020-03-03

**Authors:** Hélène Scornec, Aurore Palud, Thierry Pédron, Richard Wheeler, Clément Petitgonnet, Ivo Gomperts Boneca, Jean-François Cavin, Philippe J. Sansonetti, Hélène Licandro

**Affiliations:** ^1^PAM UMR, AgroSup Dijon, Université de Bourgogne Franche-Comté, Dijon, France; ^2^Unité de Pathogénie Microbienne Moléculaire, Institut Pasteur, Paris, France; ^3^Unité INSERM, Institut Pasteur, Paris, France; ^4^Unité de Biologie et Génétique de la Paroi Bactérienne, Institut Pasteur, Paris, France; ^5^Avenir Group, INSERM, Paris, France; ^6^Chaire de Microbiologie et Maladies Infectieuses, Collège de France, Paris, France

**Keywords:** peptidoglycan, two-component system, gene regulation, carboxypeptidase, antimicrobial peptides, lactic acid bacteria, host–microbe interaction

## Abstract

The cell surface is the primary recognition site between the bacterium and the host. An operon of three genes, LSEI_0219 (*cwaR*), LSEI_0220 (*cwaS*), and LSEI_0221 (*ldcA*), has been previously identified as required for the establishment of *Lactobacillus paracasei* in the gut. The genes *cwaR* and *cwaS* encode a predicted two-component system (TCS) and *ldcA* a predicted D-alanyl-D-alanine carboxypeptidase which is a peptidoglycan (PG) biosynthesis enzyme. We explored the functionality and the physiological role of these three genes, particularly their impact on the bacterial cell wall architecture and on the bacterial adaptation to environmental perturbations in the gut. The functionality of CwaS/R proteins as a TCS has been demonstrated by biochemical analysis. It is involved in the transcriptional regulation of several genes of the PG biosynthesis. Analysis of the muropeptides of PG in mutants allowed us to re-annotate LSEI_0221 as a putative L,D-carboxypeptidase (LdcA). The absence of this protein coincided with a decrease of two surface antigens: LSEI_0020, corresponding to p40 or msp2 whose implication in the host epithelial homeostasis has been recently studied, and LSEI_2029 which has never been functionally characterized. The inactivation of each of these three genes induces susceptibility to antimicrobial peptides (hBD1, hBD2, and CCL20), which could be the main cause of the gut establishment deficiency. Thus, this operon is necessary for the presence of two surface antigens and for a suitable cell wall architecture.

## Introduction

Advances in metagenomics have highlighted the diversity of functions encoded by the microbiota, the variability of the microbiota composition between individuals, and the link between gut microbiota composition and chronic diseases, cancer, and obesity (Arumugam et al., 2011; Lozupone et al., 2012; The Human Microbiome Project Consortium, 2012; Sonnenburg and Bäckhed, 2016; Zitvogel et al., 2017). The importance of intestinal microbiota for health is recognized, and scientific efforts are currently focused on understanding the ecological successions of microbes and microbial functions during host–microbe interactions (Gensollen et al., 2016; Milani et al., 2017; Sommer et al., 2017). Some bacterial molecules have been identified to act as signaling molecules during this interaction, such as short-chain fatty acids and CpG-DNA (cytosine-phosphate-guanine site in the single-stranded linear sequence) (Koh et al., 2016). The main components of this interaction, classed as microbe-associated molecular patterns (MAMPs), come from the bacterial cell envelope [peptidoglycan (PG), lipopolysaccharides, flagellin, teichoic acids]. They can act either in the cell wall-bound form or as released fragments for cell-to-cell signaling (Takeuchi and Akira, 2010; Clemente et al., 2012; Matos et al., 2017).

As natural inhabitants of fermented food and feed (plants, meat, dairy), as well as the oral cavity, gut, and genital tracts, *Lactobacillus* species share a good genetic arsenal to fit new and sometimes harsh environments (Makarova et al., 2006; Fiocco et al., 2019). Their high adaptability to environmental perturbations results from an accurate coordination of cellular processes (production of chaperones and DNA repair proteins, induction of metabolic pathways or transport systems, modifications of membrane composition) mediated by networks of regulators and also two-component systems (TCSs) (van de Guchte et al., 2002). *Lactobacillus casei/paracasei* is one of the best-equipped of the lactic acid bacteria (LAB) to sense and respond to environmental changes since the genome of *L. paracasei* ATCC 334 possesses 16 complete and one incomplete TCSs and 124 transcriptional regulators (Cai et al., 2009; Alcantara et al., 2011) (and our *in silico* analysis). Their resistance can also be attributed to their cell wall architecture which is the base for the maintenance of cell shape and integrity and, *via* the proteins exposed, for direct interaction with the biotic or abiotic environment (Chapot-Chartier and Kulakauskas, 2014). The cell wall of *Lactobacillus* is composed of a PG layer decorated with teichoic acids and anchored proteins like PG hydrolases and LPxTG proteins that surround the cytoplasmic membrane.

To explore the way commensal bacteria begin to colonize the gut, we have taken *L. paracasei* ATCC 334 (formerly named *L. casei* ATCC 334) as a model foodborne bacterium able to establish, at least transiently, in the gut and interact with the host (Licandro-Seraut et al., 2014). *L. paracasei* is one of the most studied LAB species in food microbiology, particularly for its flavoring abilities (Di Renzo et al., 2018; Stefanovic et al., 2018) and for its probiotic properties (Arioli et al., 2018; Fehlbaum et al., 2019). Using signature-tagged mutagenesis coupled with screening in a ligated rabbit ileal-loop model, we have identified a core of 47 genes in *L. paracasei* essential for gut establishment, the first step of colonization. Indeed, five genes could be attributed to adaptation to environment (three regulators and one TCS—predicted) and six genes to biogenesis of the cell wall [three genes implicated in D-alanylation of lipoteichoic acids (LTAs), two transporters, and one D-alanyl-D-alanine carboxypeptidase—predicted]. Among them, three consecutive, identically oriented genes were identified: *LSEI_0219*, *LSEI_0220*, and *LSEI_0221*. The *LSEI_0219–0220* genes caught our attention since they encode the only TCS identified in this screening. Also, *LSEI_0221* is the only gene annotated as a putative D-alanyl-D-alanine (D-Ala-D-Ala) carboxypeptidase, penicillin-binding protein (PBP) in *L. paracasei* (Cai et al., 2009). Genetic location of *LSEI_0219–0220* presumes a role of this TCS in the cell wall biogenesis. In light of the results reported hereafter, *LSEI_0219*, *LSEI_0220*, and *LSEI_0221* genes were named *cwaR*, *cwaS*, and *ldcA*, respectively. In this study, we explored biochemical functions and physiological roles of *cwaR*, *cwaS*, and *ldcA* genes and their corresponding proteins. We also assessed the consequences of their inactivation, which may explain the defect in surviving in the gut previously observed.

## Materials and Methods

### Bacterial Strains, Plasmids, and Growth Conditions

Bacterial strains and plasmids used in this study are listed in Table 1. *L. paracasei* ATCC 334 and mutants were grown statically at 37°C in MRS medium (Difco), supplemented with 5 μg.ml^–1^ erythromycin for mutants. The following mutants, M*cwaR*, M*cwaS*, M*ldcA*, M*dltA*, M*dltC*, and *dltD*, were obtained by transposon mutagenesis with P_junc_-TpaseIS*_1223_* and identified by individual sequencing as previously described (Licandro-Seraut et al., 2012, 2014; Scornec et al., 2014). *Escherichia coli* strains TG1 and BL21(DE3) were used as cloning and expression hosts, respectively. They were grown in LB medium at 37°C with shaking. Recombinant plasmids in *E. coli* were selected in LB medium containing 50 μg.ml^–1^ kanamycin.

**TABLE 1:** Bacterial strains and plasmids.

Strains and plasmids	Genotype and/or relevant feature(s)	Source or reference
**Strains**		
*L. paracasei* ATCC 334	Wild type, CIP 107868, genome sequenced	Collection Institut Pasteur, France
M*cwaR mutant*	ATCC 334 LSEI_0219*:*pVI110, Erm^R^	Licandro-Seraut et al., 2014
M*cwaS* mutant	ATCC 334 LSEI_0220*:*pVI110, Erm^R^	Licandro-Seraut et al., 2014
M*ldcA* mutant	ATCC 334 LSEI_0221*:*pVI110, Erm^R^	Licandro-Seraut et al., 2014
M*dltA* mutant	ATCC 334 LSEI_0794*:*pVI110, Erm^R^	Licandro-Seraut et al., 2014
M*dltC* mutant	ATCC 334 LSEI_0796*:*pVI110, Erm^R^	Licandro-Seraut et al., 2014
M*dltD* mutant	ATCC 334 LSEI_0797*:*pVI110, Erm^R^	Licandro-Seraut et al., 2014
*E. coli* TG1	*SupE hsd*Δ*5thi*Δ*(lac-proAB)*F’ *[tra D36 pro AB^+^ lac I^*q*^ lacZ*Δ*M15]*	Gibson, 1984
*E. coli* BL21 (DE3)	F^–^ *ompT hsdS_*B*_* (rB^–^mB^–^) *gal dcm* (DE3)	Invitrogen
*E. coli* TG1 pET*cwaR*	*SupE hsd*Δ*5thi*Δ*(lac-proAB)*F’ *[tra D36 pro AB^+^ lac I^*q*^ lacZ*Δ*M15]* with pET*cwaR*	This work
*E.coli* TG1 pET*ccwaS*	*SupE hsd*Δ*5thi*Δ*(lac-proAB)*F’ *[tra D36 pro AB^+^ lac I^*q*^ lacZ*Δ*M15]* with pET*ccwaS*	This work
**Plasmids**		
pET28a+	Kan^R^, vector for overexpression of His-tagged proteins using the T7 bacteriophage promoter	Novagen
pET*cwaR*	pET28a + containing LSEI_0219 gene between *Nco*I and *Xho*I sites to overproduce CwaR His_6_ tag	This work
pET*ccwaS*	pET28a + containing truncated LSEI_0220 gene coding for residues 137-396 of the HK protein between *Nco*I and *Xho*I sites to overproduce cCwaS His_6_ tag	This work

### DNA Techniques

All DNA manipulations were performed according to standard procedures (Sambrook et al., 1989). Plasmids were isolated by using Nucleospin plasmid miniprep kit (BioBasics). PCR was performed using 0.02 unit of Phusion High-Fidelity DNA polymerase (Thermo Scientific).

### Stress Conditions for qRT-PCR Analysis

For 15-min stresses, culture in exponential phase [OD at 600 nm (OD) = 0.6] was suspended in a volume of MRS supplemented with NaCl 1 M, bile 3 g/l, acid (HCl) pH 3.0, lactic acid pH 3.0, penicillin 0.1 μg/ml, vancomycin 0.5 g/l, or ethanol 15% (v/v) (Palud et al., 2018). For thermal stresses, phosphate buffer (20 mM pH 6.0) at 4 or 50°C was used instead of MRS. For sodium dodecyl sulfate (SDS) treatment, phosphate buffer was supplemented with SDS 0.05% (*m/v*). MRS or phosphate buffer was used as the reference.

### RNA Extractions and qRT-PCR Analysis

PG-related genes were selected based on *L. paracasei* ATCC 334 genome annotation (NCBI annotation number NC_008526.1 and NC_008502.1).

Whole RNA was extracted from 50 ml of culture in exponential phase (OD = 0.6) and 10 ml in stationary phase (OD = 3.5) after bead beating disruption using Tri reagent method (Sigma), and cDNA were synthesized as previously described (Licandro-Seraut et al., 2008). Quantitative reverse transcriptase PCRs (qRT-PCR) were performed in a CFX384 real-time detection system (Bio-Rad). The total volume of the PCR mixture was 15 μl containing 1X SsoAdvanced^TM^ Universal SYBR^®^ Green Supermix (Bio-Rad), 25 ng cDNA, and 0.8 μM of each primer (Supplementary Table S1). PCR amplification was one step at 95°C for 30 s followed by 40 cycles of 95°C for 5 s and 60°C for 10 s. An additional cooling step from 90°C to 60°C (3°C/min) was performed to establish a melting curve in order to verify the homogeneity of the amplicon. All sample and primer combinations were assessed in three biological replicates with at least two technical replicates per biological replicate. In each run, a negative control using sterile water instead of cDNA was included. Normalized expression levels were calculated using the comparative critical threshold (ΔΔCq) method (Hellemans et al., 2007). The data were processed with CFX Manager Software (Bio-Rad).

### Plasmid Constructs and Generation of *E. coli* Strains Overproducing RR0219 and cHK0220

Full-length *cwaS* was amplified using primers LSEI_0219FbisNcoI (5′-ATACCATGGGCAAAATTTTAATTGTTGA-3′) and LSEI_0219RXhoI (5′-GTGCTCGAGGGCCTCAACCTTATAGCCGA-3′), and the cytosolic domain of CwaS, cCwaS (residues 137 to 396) was amplified using primers LSEI_0220FHAMPNcoI (5′-ATACCATGGGCACCGTCAATTCAATGGCC-3′) and LSEI_0220RXhoI (5′-GTGCTCGAGTTTTGACTTTCCTGCTTCCT-3′) from the *L. paracasei* genomic DNA. The forward primers contained the *Nco*I site in frame with the translation start codon, and the reverse primers contained the *Xho*I site. After restriction digestion, the amplicons were ligated to *Nco*I-*Xho*I digested pET28a+ expression vector using T4 DNA ligase HC (Fermentas). The resulting ligation products were directly transformed by electroporation into the *E. coli* TG1 strain as described by Dower et al. (1988) to obtain pETCwaR and pETcCwaS constructs. Plasmids were isolated from selected transformants and subjected to sequencing reactions (GATC Biotech). The pETCwaR and the pETcCwaS were transformed into competent *E. coli* BL21 (DE3) cells by the CaCl_2_ procedure (Cohen et al., 1972), and protein expression was induced at OD ≈ 0.5 in 1 L of LB with the addition of 0.5 mM IPTG for 3 h at 37°C.

### Cell Extracts and Protein Purification

*Escherichia coli* or *Lactobacillus paracasei* cell extracts were prepared as previously described (Gury et al., 2009). For protein purification, the filtrates were applied to a Protino^®^ Ni-NTA column (Macherey-Nagel), and the CwaR and cCwaS proteins were eluted with imidazole gradient.

### Autophosphorylation and Phosphotransfer Assay

Immediately before use, 0.5 ml of purified CwaR and cCwaS proteins were concentrated using an ultrafree 0.5 centrifugal filter unit (Millipore). The autophosphorylation procedure was adapted from Abo-Amer et al. (2004). Briefly, 6 μg of cCwaS protein was incubated in a final volume of 20 μl phosphorylation buffer containing 50 mM Tris-HCl pH 7.5, 2.5 mM MnCl_2_, 5 mM MgCl_2_, 50 mM KCl, 20 μCi of [γ-^32^P]-ATP (3,000 Ci/mmol). Reaction was stopped by addition of 4 μl SDS loading buffer. For the phosphotransfer reaction, 10 μg of CwaR protein was added after 60 min of cCwaS protein autophosphorylation, and samples were quenched by the addition of 5 μl SDS loading buffer. The reaction mixtures were analyzed by 12.5% SDS-polyacrylamide gel electrophoresis (SDS-PAGE), followed by autoradiography.

### Phosphorylation of CwaR Protein by Small-Molecule Phosphoryl Donors

The phosphorylation by acetyl phosphate protocol was adapted from Molle and Buttner (2000) and Hutchings et al. (2006). [^32^P]-acetyl phosphate was prepared by incubating acetate kinase (0.4 units; A7437, Sigma) in 55 μl of 25 mM Tris-HCl pH 7.5, 60 mM potassium acetate, 10 mM MgCl_2_, 100 μCi [γ-^32^P]-ATP (3,000 Ci/mmol) for 30 min at 25°C. The [^32^P]-acetyl phosphate solution was mixed with 40 μl of 50 mM Tris-HCl pH 7.5, 2.5 mM MnCl_2_, 5 mM MgCl_2_, and 50 mM KCl containing 40 μg of CwaR protein. After incubation at 37°C, samples of 18 μl were quenched by addition of 2 μl of SDS loading buffer and analyzed by a 12.5% SDS-PAGE, followed by autoradiography. RR0219 protein (3 μg) was incubated with 60 mM of acetyl phosphate 50 mM Tris-HCl pH 7.5, 2.5 mM MnCl_2_, 5 mM MgCl_2_, and 50 mM KCl.

### Electrophoretic Mobility Shift Assays

Electrophoretic mobility shift assays (EMSAs) were carried out on cytosolic protein extract as previously described with some modifications (Gury et al., 2004). The 443-pb DNA probe, named Prom*cwaR*, corresponding to the whole LSEI_0218–LSEI_0219 intergenic region was PCR amplified with primers P0219F-189 (5′-GCTAAGGCTTTTGTCAATATGG-3′) and P0219R254 (5′-CTTAGTAATTCGACGATTTC-3′). The fragment was 3′ end-labeled with digoxigenin (DIG)-11-ddUTP with dig gel shift kit, second generation (Roche) according to the manufacturer’s recommendation. Before binding reactions, protein extract was heated for 10 min at 65°C, then it was incubated with 1 μl of 1/500 Prom*cwaR* probe in 15 mM Tris-HCl pH 7.5, 5% glycerol (v/v), 2 mM EDTA, 50 mM NaCl, 25 mM KCl, 2.5 mM MgCl_2_, 2 mM DTT, 2.5 μg.ml^–1^ of bovine serum albumin (BSA) and 2.5 μg.ml^–1^ of salmon sperm DNA as an unspecific DNA competitor in a 15-μl reaction volume for 20 min at 30°C. The samples were resolved onto 5% (w/v) PAGE gel in 20 mM Tris-HCl, pH 7.8, 400 mM glycine, and 1 mM EDTA at 4°C. The DNA was transferred to a nylon membrane by electroblotting, probed with anti-DIG-AP antibody (Roche), and visualized using chemiluminescence.

### Transmission Electron Microscopy

Cells were pelleted by centrifugation and fixed overnight at 4°C in 3% glutaraldehyde (v/v), 2% formaldehyde (v/v), 5 mM MgCl_2_, and 0.1 M phosphate buffer, pH 7.2. Then, cells were pelleted and embedded in 2.25% (w/v) low-gelling temperature agarose. Samples were fixed in 0.5% OsO_4_ (m/v) in 0.1 M phosphate buffer pH 7.2 for 1 h at 4°C, then dehydrated through graded concentrations of ethanol then propylene oxide, and embedded in Epon 812 (Merck, Lyon, France). Ultrathin sections were stained with PATag method as described previously (Thiéry, 1967) and observed at 80 kV on a transmission electron microscope (H-7500, Hitachi, Tokyo).

### Peptidoglycan Extraction and Analysis

*Lactobacillus paracasei* ATCC 334 and the M*ldcA* mutant were purified and analyzed as described by de Jonge et al. (1992) with some modifications (Girardin et al., 2003). The traces are representative data of the three replicates.

### Minimal Inhibitory Concentration Determination

The minimal inhibitory concentrations (MICs) of penicillin, bacitracin, nisin, and vancomycin against *L. paracasei* ATCC 334 and M*ldcA* mutant were determined by a microdilution technique. Overnight cell cultures were diluted 1:100 in fresh MRS media and added in individual wells containing various concentrations of antibiotic (series of twofold dilutions). The MIC was defined as the lowest antimicrobial concentration preventing visible turbidity after 24 h of incubation at 37°C.

### Microbial Adhesion to Solvents Test

A Microbial Adhesion To Solvents (MATS) test was performed as described previously (Bellon-Fontaine et al., 1996) with chloroform (acidic solvent and electron acceptor), ethyl acetate (basic solvent and electron donor), and hexadecane (hydrophobic solvent). Overnight cultures of each strain were washed and suspended at OD = 0.4 in phosphate-buffered saline (PBS, pH 7.4) (A_0_). This suspension (3 ml) was mixed with 1 ml of solvent (chloroform, ethyl acetate, or hexadecane) for 3 min with vortex. After 15 min (partition of phases), the OD of the aqueous phase was measured (A_1_). The percentage of adhesion was calculated as follows:% adhesion = 100 × ((A_0_-A_1_)/A_0_). All experiments were repeated at least three times on three biological repeats.

### Triton X-100-Induced Autolysis

*Lactobacillus paracasei* strains were grown on MRS medium to mid-exponential phase (OD = 0.8). Cells were harvested by centrifugation, washed, and then suspended to OD of 1.0 in a 50 mM potassium phosphate buffer, pH 7.0, supplemented with 0.05% Triton X-100 (v/v). Cell suspensions were then transferred in 96-well sterile clear microplates (Nunc) and incubated 24 h at 30°C under agitation. Autolysis was monitored by measuring the OD. All experiments were repeated at least three times on three biological repeats.

### Surface Protein Extraction and Identification

Surface proteins were extracted by 5 M of LiCl according to the Smit et al. (2001) procedure (Zhang et al., 2010). Briefly, 20 ml of bacterial cultures were washed twice with ice distilled water. The cell pellets were suspended in 2 ml of 5 M of LiCl and incubated 1 h at 37°C with shaking. Suspensions were centrifuged 10,000 *g* at 10°C for 20 min, then supernatants were precipitated with 4 ml of cold acetone and incubated for 2 h at −20°C. The precipitated proteins were harvested by centrifugation (10,000 *g*, 10 min at 4°C). The precipitates were suspended in loading SDS buffer then boiled 3 min and analyzed by 12.5% SDS-PAGE. Briefly, for protein identifications, proteins were reduced, alkylated, then digested by trypsin (Promega) for 3 h at 37°C. Tryptic digests were separated and analyzed by liquid chromatography coupled to tandem mass spectroscopy (LC-MS/MS) (nanoLC-ESI-Trap, Orbitrap ELITE, Thermo Scientific). Protein identification was performed using Proteome Discoverer 1.4 software against a protein database of *L. paracasei* ATCC 334 strain (GenBank: NC_008526.1 and NC_008502.1).

### Challenge in Stressful Conditions

Overnight cultures were combined to get a proportion of 2% of mutant and 98% of ATCC 334. The resulting mixed cultures were washed with PBS pH 7.4, suspended to a final OD of 0.1 and challenged during 16 h at 37°C in diluted MRS (1/20), used as a low-nutrient medium, and modified to obtain different stressful conditions: (i) low-nutrient, acidic (HCl) (pH 3.5), (ii) low-nutrient, bile supplemented (3 g.l^–1^), (iii) low-nutrient, lysozyme-supplemented (10 g.l^–1^). The concentrations of mutant relative to ATCC 334 were determined by counting (CFU.ml^–1^) on MRS + Ery 5 μg.ml^–1^ and MRS plates. Mutant resistance to stress was assessed by determining the ratio of mutant to the ATCC 334 strain and comparing it to the initial ratio. The ratio with the control treatment (MRS diluted 1/20) is the same before and after 16 h at 37°C.

### Susceptibility to Cationic Peptides

Cationic peptide susceptibility tests were performed as previously described (Hugo et al., 2012). Bacterial cultures (50 μl) diluted in 50 mM *N*-2-hydroxyethylpoperazine-N9-2-ethanesulfonic acid (Hepes) buffer pH 7.5 at 4.10^7^ CFU.ml^–1^ were incubated with 50 μl of hBD1, hBD2, LL37, or CCL20 (Abcam) at 20 μg.ml^–1^ in Hepes buffer for 1 h at 37°C with 5% CO_2_. Treated and non-treated bacteria were then incubated for 5 min at room temperature with 400 μl of propidium iodide (PI) at 5 μg.ml^–1^. The ratio of PI(+) cells was assessed by flow cytometry (FACSCanto, Diva software; Becton Dickinson, Mountain View, CA, United States). For each sample, 10,000 events were analyzed.

### Statistical Analyses and Software

Data are expressed as arithmetic means ± SD. The statistical analyses and the software used are indicated in the figure legends. Significant differences are indicated by (^∗^) in the figures.

## Results

### Predictive Analysis of Operon *cwaRS-ldcA* and Polar Effect of the Transposon Insertion

**FIGURE 1:**
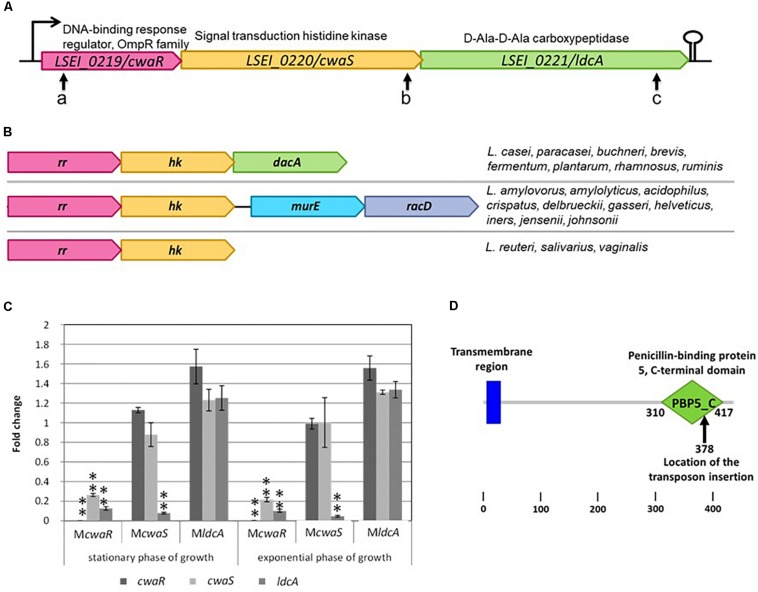
Description of *cwaRS-ldcA* locus. **(A)** Locations of the different transposon insertions are shown with arrows a, b, and c for mutants M*cwaR*, M*cwaS*, and M*ldcA*, respectively. **(B)** Comparison of the organization in different *Lactobacillus* species. *rr*, response regulator gene; *hk*, histidine kinase gene. **(C)** Relative transcript levels of *L. casei* mutants in stationary and exponential phase of growth in MRS broth. Transcript levels of each gene are expressed as the relative fold change, with *L. paracasei* ATCC 334 as the reference condition (fold change = 1). Three biological repeats were performed, and bars indicate standard deviations. Statistical analysis was performed using the unpaired Student *t-*test: ^∗^*P* < 0.01; ^∗∗^*P* < 0.001. **(D)** Smart schema of the domains within LdcA protein and location of the transposon in mutant M*ldcA*.

The *cwaRS-ldcA* predicted operon is composed of three genes (Figure 1A). The *cwaR* gene (*LSEI_0219*) belongs to the OmpR family and is a predicted response regulator (RR); the *cwaS* gene (*LSEI_0220*) is a predicted signal transduction histidine kinase (HK); the *ldcA* gene (*LSEI_0221*) is a predicted D-Ala-D-Ala carboxypeptidase (penicillin-binding protein). This family of TCS is widely encountered in the bacterial world. In lactobacilli, it can be found in three different genetic organizations (Figure 1B). The TCS genes can be followed by a D-Ala-D-Ala carboxypeptidase as in *L. paracasei* or by another putative operon composed of *murE* (UDP-*N*-acteylmuramoylalanyl-D-glutamate-2,6-diaminopimelate ligase) and *racD* (aspartate racemase). In a few species, the locus is only composed of the TCS predicted operon. The first and the third configurations are observed in vancomycin-resistant *Lactobacillus* species (Mackey et al., 1993; Wilks et al., 2004; Klare et al., 2007; Balandino et al., 2008) except for *L. fermentum* which displays a variable strain-dependent vancomycin resistance (Rossetti et al., 2009). The second configuration is only observed in the vancomycin-susceptible species.

The coding sequences of *cwaR* and *cwaS* are separated by only five nucleotides, and *cwaS* and *ldcA* partially overlap. Thus, we assumed that these three genes were co-transcribed, which was confirmed by RT-PCR (data not shown). To determine if transposon insertion in the operon triggers a polar effect, qRT-PCR was performed for the three genes in the three corresponding mutants during exponential and stationary phases of growth (Figure 1C). Transcripts corresponding to *cwaS* and *ldcA* in *cwaR* mutant (M*cwaR*, pVI110 insertion in *cwaR* gene) and transcripts corresponding to *ldcA* in *cwaS* mutant (M*cwaS*, pVI110 insertion in *cwaS* gene) were at least fivefold lower than in the ATCC 334 parental strain, meaning that transcription is affected downstream of the transposon insertion site. Also, transcript levels corresponding to genes upstream of the transposon insertion in these two mutants (M*cwaS* and M*ldcA*) were similar to the ATCC 334, meaning that transposon insertion does not affect the stability of the upstream transcripts. In M*ldcA* mutant, the transcript for the *ldcA* gene is not affected by insertional inactivation because the transposon insertion site is rather at the end of the *ldcA* gene, but this locus corresponds to the middle of the predicted PBP5-C domain (Figure 1D) (Letunic et al., 2014). Thus, the protein function must be inactivated in this mutant. One possible assumption was that the defect of these three mutants in gut establishment could result from the inactivation of activity or the transcriptional decrease of *ldcA* only.

### Physiological Role of D-Ala-D-Ala Carboxypeptidase for *L. paracasei*

**FIGURE 2:**
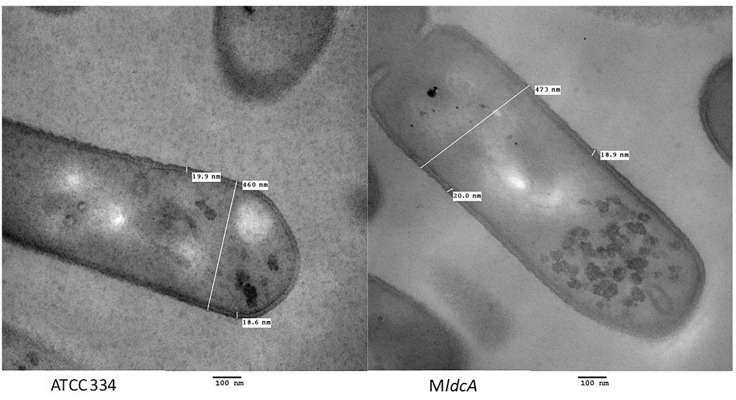
Transmission electron microscopy micrographs of *L. paracasei* ATCC 334 and *LSEI_0221* mutant grown in MRS medium until the early stationary phase of growth.

The *ldcA* gene encodes a predicted D-Ala-D-Ala carboxypeptidase, a hydrolase involved in PG maturation which cleaves the pentapeptide side chain of PG strand between the fourth and the fifth (and last) alanine residues, thereby allowing transpeptidation of two PG strands. For *L. casei/paracasei*, the last D-alanine residue is substituted by a D-lactate, conferring vancomycin resistance (Handwerger et al., 1994; Billot-Klein et al., 1997). This function is essential for PG synthesis and, since no other *L. paracasei* genes are predicted to encode this enzyme, *ldcA* inactivation should be lethal. However, the M*ldcA* mutant is viable and grows as well as the ATCC 334 in culture medium (Licandro-Seraut et al., 2014). In view of this, it is likely that the annotation is not correct: the enzyme probably has another activity and there is another enzyme with the activity D-Ala-D-Ala carboxypeptidase like LSEI_0141 (annotated as a microcin self-immunity protein in ATCC 334 strain and as containing a peptidase domain with D,L carboxypeptidase activity in other *L. casei* genomes) and LSEI_2553 (uncharacterized but annotated as containing a peptidase domain which has a possible carboxypeptidase activity). Thus, we decided to test whether *ldcA* truly encoded the predicted function. Transmission electron microscopy (TEM) analysis of the M*ldcA* mutant cultivated in exponential and early stationary phases of growth demonstrated that its cell morphology was similar to that of the ATCC 334 with length between 1 and 2 μm and width between 0.4 and 0.6 μm (Figure 2). Moreover, its PG thickness was not altered in the mutant with a value of 19.4 ± 3.6 nm.

**FIGURE 3:**
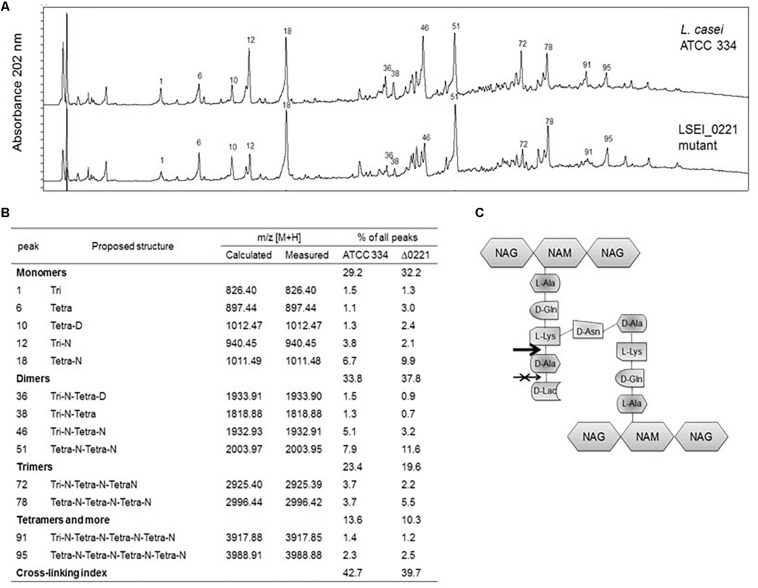
Muropeptide profiles of *L. paracasei* ATCC 334 and *lcdA* mutant. **(A)** Reversed phase high-performance liquid chromatography (RP-HPLC) separation profiles of muropeptides of *L. paracasei* and *lcdA* mutant, peptidoglycans were digested by mutanolysin. Numbers correspond to the main muropeptides whose amounts changed between ATCC 334 and *lcdA* mutant. **(B)** Main muropeptide compositions, Tri, disaccharide tripeptide (L-Ala-D-iGln-L-Lys); Tetra, disaccharide tetrapeptide (L-Ala-D-iGln-L-Lys-D-Ala); Disaccharide, GlcNAc-MurNAc; iGln, isoglutamine; *N*, D-Asn; D, D-Asp. Percentage of each peak was calculated as the ratio of the peak area to all peak areas. Cross-linking index was calculated according to Glauner (1988) with the formula (1/2 Σ dimers + 2/3 Σ trimers + 3/4 Σ tetramers and more)/Σ all muropeptides. **(C)** Schematic structure of *L. paracasei* ATCC 334 peptidoglycan. The arrow indicates the cleavage site of LdcA enzyme, and the crossed arrow indicates the incorrect, predicted function.

To assign a biochemical function to the enzyme encoded by *ldcA* gene, an analysis of muropeptides was performed on M*ldcA* mutant and ATCC 334 strains. The reversed phase high-performance liquid chromatography (RP-HPLC) muropeptide profiles revealed that the PG composition of M*ldcA* mutant differs from that of ATCC 334: tripeptides (peaks 1, 12, 36, 38, 46, 72, 91) are less represented while tetrapeptides (peaks 6, 10, 18, 51, 78, 95) are more represented. However, the PG cross-linking index was approximately the same (Figures 3A,B). These results suggest an L,D-carboxypeptidase activity, which cleaves the peptide chain of PG between L-Lys and D-Ala, rather than the predicted D,D-carboxypeptidase activity (Figure 3C). Thus, the LSEI_0221 gene was named *ldcA* for its putative L,D-carboxypeptidase activity. The M*cwaR* and M*cwaS* mutants presented the same profiles as the M*ldcA* mutant (data not shown).

### Consequences of the Peptidoglycan Composition on the Surface Properties

The PG composition differences between M*ldcA* mutant and ATCC 334 could impact surface properties that are essential for antibiotics and autolysis resistances and for biofilm formation (Perpetuini et al., 2016). Thus, these properties were evaluated for the M*ldcA* mutant and compared to the ATCC 334.

**FIGURE 4:**
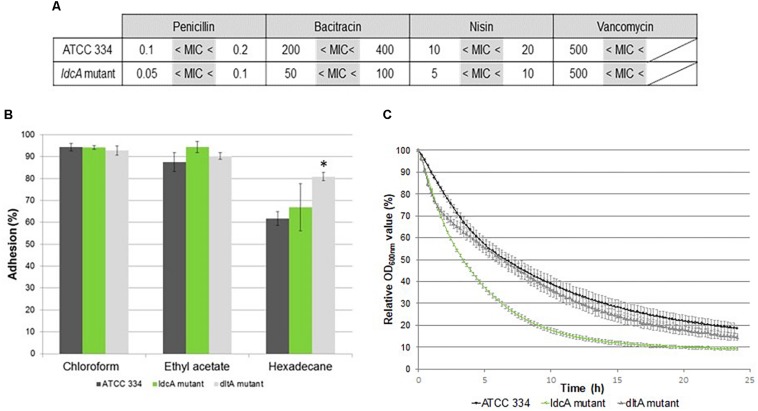
Analysis of surface properties of ATCC 334 and *ldcA* mutant of *L. paracasei.* **(A)** Minimal inhibitory concentration (MIC) of ATCC 334 and the *ldcA* mutants in μg.ml^–1.^ **(B)** Microbial Adhesion To Solvents (MATS) tests of ATCC 334 and the *ldcA* and *dltA* mutants. Error bars represent standard deviations between three biological repeats. Statistical analysis was performed for each mutant versus ATCC334 using the unpaired Student *t* test: ^∗^*P* < 0.01. **(C)** Autolysis profiles of ATCC 334 and the *ldcA* and *dltA* mutants. Cultures were grown to the exponential phase of growth (OD of 0.8), then the cells were pelleted and suspended in phosphate-buffered saline (PBS) containing 0.05% Triton X-100 (v/v) and incubated at 30°C. Error bars represent standard deviations between three biological repeats.

The MICs of four antimicrobials (penicillin, bacitracin, nisin, and vancomycin), which inhibit PG biogenesis at different steps, were determined (Figure 4A). As the M*ldcA* mutant was not more susceptible to vancomycin than the ATCC 334, we concluded that the altered PG of the mutant still contained pentadepsipeptides ending with D-Ala-D-Lac (Handwerger et al., 1994; Billot-Klein et al., 1997). On the contrary, the mutant displayed an increased susceptibility to penicillin, bacitracin, and nisin. The increased susceptibility to penicillin corresponded to the expected phenotype for a PBP defective mutant. The increased bacitracin susceptibility suggested a slower renewal of PG since this antibiotic inhibits undecaprenyl phosphate regeneration, an indispensable step to synthesize new PG strains. The nisin acts as a membrane disturber. The reason why M*ldcA* mutant was more susceptible to nisin than ATCC 334 could be an easier passage of nisin through the cell wall, most likely due to a change in surface polarity and/or by a slower renewal of PG (nisin binds lipid II and inhibits PG synthesis).

Thus, bacterial surface properties were assessed by MATS tests on the M*ldcA* mutant, ATCC 334, and M*dltA* mutant (*LSEI_0794*). The M*dltA* mutant is altered in teichoid acid D-alanylation, a function that contributes to reduce the negative net charge of the cell wall surface (Neuhaus and Baddiley, 2003). The MATS values indicate that *L. paracasei* ATCC 334 surface displays basic, acidic, and hydrophobic properties in agreement with previous observations (Munoz-Provencio et al., 2009) (Figure 4B). The M*dltA* mutant displays a higher surface hydrophobicity than ATCC 334. Surface properties of the M*ldcA* mutant are the same as the ATCC 334. Thus, PG modifications in the M*ldcA* mutant do not impact surface properties and do not seem to alter teichoic acid presentation at the cell surface.

Autolysis profiles were assessed by exposing bacterial cells to Triton X-100, a detergent known to remove the LTA. LTA acts as an inhibitor on the general bacterial autolytic system (endogenous autolysins) (Raychaudhuri and Chatterjee, 1985). As a *Lactobacillus rhamnosus* GG Δ*dltD* mutant has been reported to display an increased autolysis (Perea Velez et al., 2007), the *L. paracasei* M*dltA* mutant was also submitted to this autolysis test for comparison. The M*ldcA* mutant exhibited higher Triton X-100-induced autolysis rates than the ATCC 334 strain (Figure 4C). This autolysis rate was similar to the M*dltA* mutant during the first 2 h and was even higher after. This observation suggested either a higher recruitment of autolysins in this mutant, despite a correct teichoic acid presentation, or the structure of the PG renders it more susceptible to autolysin activity.

### Functionality of the CwaRS TCS *in vitro*

**FIGURE 5:**
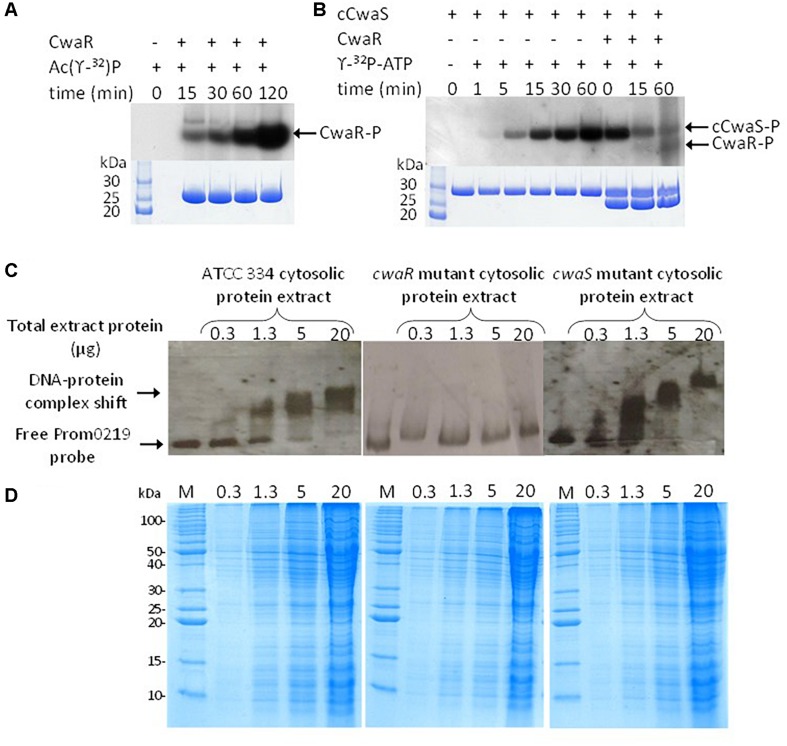
*In vitro* characterizations of the two-component system (TCS). **(A)** Radiolabeling of RR0219 using [γ -^32^P] acetyl phosphate after different times of incubation. **(B)** Autophosphorylation of the cytosolic domain of HK0220 (cHK0220) when incubated with [γ -^32^P]ATP. After an incubation of 60 min, the RR0219 protein was added, and the phosphotransfer from radiolabeled cHK0220 (cHK0220-P) to RR0219 was observed after different times of co-incubation. **(C)** Electrophoretic mobility shift assay (EMSA) of *cwaR* promoter with cellular extract of *L. paracasei* ATCC 334 and the *cwaR* mutant. **(D)** The corresponding sodium dodecyl sulfate–polyacrylamide gel electrophoresis (SDS-PAGE) is 12.5%.

The *cwaR* gene was overexpressed in *E. coli*, and the resulting CwaR protein was purified. CwaR size analyzed by SDS-PAGE was consistent with that expected (27 kDa) (Figure 5A). Incubation of purified CwaR with ^32^P radiolabeled acetyl-phosphate showed that CwaR was able to autophosphorylate using acetyl phosphate, a molecule which is a common phosphate donor (Figure 5A). The cytosolic domain of CwaS, named cCwaS, corresponding to residues 137–396 of the CwaS protein, was overexpressed in *E. coli*, purified and a 29-kDa protein was obtained. It was able to autophosphorylate using [γ-^32^P]-ATP as the phosphate donor which confirms the predicted kinase function (Figure 5B). To visualize if CwaS-R can function together as a TCS *in vitro*, the radiolabeled phosphorylated cCwaS was incubated with the purified CwaR. A partial dephosphorylation of the cCwaS and a phosphorylation of the CwaR were visible, demonstrating that the cCwaS was able to transfer phosphate to the CwaR (Figure 5B). The loss of the ^32^P from cCwaS-P was much greater than the gain of ^32^P on CwaR, but it is an *in vitro* experiment and the phosphorylation rate is not 100%. The predicted binding phosphate residues are His163 for CwaS and Asp52 for CwaR (determined by *in silico* analysis).

EMSAs were carried out with the promoter of the *cwaRS-ldcA* operon for two purposes. The first was to complete the demonstration of the functionality of the TCS *in vitro.* The second was to determine whether the TCS satisfies the observation that, when part of an operon, it is generally able to act on its own promoter and to autoregulate the whole operon (Stock et al., 2000). The phosphorylated and unphosphorylated forms of the purified CwaR protein were incubated individually with a 443-bp probe, named Prom*cwaR*, corresponding to the whole *LSEI_0218-cwaR* intergenic region for EMSA. Despite all our attempts using different experimental conditions (binding buffer, freshly prepared CwaR, phosphorylated or unphosphorylated, …), no interaction between the Prom*cwaR* probe and the purified RR could be observed (data not shown). The failure of EMSA with the purified CwaR could be due to CwaR misfolding during protein production, to non-adapted buffer conditions, or the lack of a co-factor or protein partner. Thus, EMSAs were carried out using crude cytosolic protein extracts of *L. paracasei* ATCC 334 (containing both CwaS and CwaR proteins), of M*cwaR* mutant (containing CwaS—probably at a smaller level than ATCC 334 because of the alteration of transcription demonstrated before—but not CwaR) and of the M*cwaS* mutant (containing CwaR but not CwaS protein) (Figure 5C). The Prom*cwaR* probe was shifted when protein extracts from ATCC 334 and the M*cwaS* mutant were used, but not by those from the M*cwaR* mutant, for the same cytosolic proteins concentration (Figure 5D). Thus, CwaR is able to bind the promoting region of the operon in conditions that could not be replicated *in vitro* with the purified CwaR. It acts as the transcriptional regulator of its operon, and it needs some additional cellular elements and/or a protein co-factor to be active at this DNA site.

### Consequences of *cwaR*, *cwaS*, and *ldcA* Gene Inactivation on the Regulation of Genes Involved in Peptidoglycan Synthesis

**FIGURE 6:**
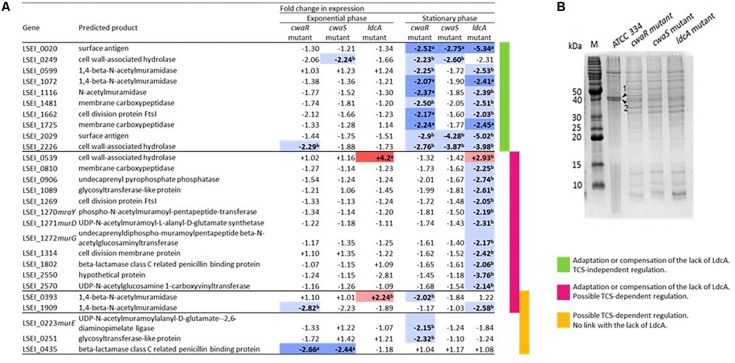
Consequences of *cwaRS* gene inactivation on genes encoding in peptidoglycan (PG) synthesis proteins and surface proteins. **(A)** Transcriptional profiles for genes implicated in PG synthesis of the M*cwaR*, M*cwaS*, and M*ldcA* mutants relative to the parental strain ATCC 334 grown either exponential or stationary phase. Values are the mean RTL obtained for three biological repeats. In bold letters, significant values; in red and ^a^, upregulation, *p* < 0.01; in light red and ^b^, upregulation, *p* < 0.05; in blue and ^a^, downregulation, *p* < 0.01; in light blue and ^b^, downregulation, *p* < 0.05. Genes for which no significant value was obtained are not presented. The list of the tested genes and their corresponding annotations are presented in Supplementary Table S2. **(B)** Sodium dodecyl sulfate–polyacrylamide gel electrophoresis (SDS-PAGE) of surface protein extracts from *L. paracasei* ATCC 334 and M*cwaR*, M*cwaS*, and M*ldcA* mutants in stationary phase of growth. (1) Surface antigen (LSEI_2029), (2) Surface antigen (LSEI_0020).

Transcriptomic analysis of 50 PG-related genes in M*cwaR*, M*cwaS*, and M*ldcA* mutants compared to ATCC 334 was carried out in exponential and stationary phases of growth for two main purposes: (i) determining if the lack of LdcA was partly compensated by another enzyme and (ii) identifying the possible PG-related genes regulated by the CwaRS TCS. Only two genes were upregulated in the M*ldcA* mutant: *LSEI_0393* (for exponential phase only) and *LSEI_0539* (Figure 6A and Supplementary Table S2). *LSEI_0539* encodes a cell wall-associated hydrolase with an NLPC-P60 and a glucosamidase domain; it could act as an *N*-acetylglucosamidase or an endopeptidase (Layec et al., 2008). *LSEI_0393* encodes a 1,4-beta-*N*-acetylmuramidase with a glyco_hydro_25 and a LysM domain (Layec et al., 2008). These proteins may bring functional compensation of the LdcA absence. These upregulations could explain the higher rate of autolysis in mutant. Overall, only a few PG-related genes were differentially transcribed in exponential phase, whereas many of these genes were downregulated in the stationary phase of growth in the M*cwaR* mutant, and even more in M*ldcA* mutant, as though gene regulation was directed to slow down the turnover of PG. The strongest downregulated genes in all mutants were *LSEI_0020*, *LSEI_2029*, and *LSEI_2226* (Figure 6A). *LSEI_2029* and *LSEI_2226* encode predicted cell wall-associated hydrolases. The downregulation of genes in the stationary phase was generally less important in McwaS mutant than in McwaR mutant, suggesting that the regulator responds to other sensors. By searching the differences of relative transcript levels between M*cwaR* mutant (inactivated TCS and lack of LdcA) and M*ldcA* mutant (functional TCS and inactivated LdcA), we propose 17 candidate genes to be regulated by the CwaRS TCS. They can be split into two subgroups: (i) those that are differently expressed in the M*ldcA* mutant (*LSEI_0539*, *LSEI_0810*, *LSEI_0906*, *LSEI_1089*, *LSEI_1269-1272*, *LSEI_1314*, *LSEI_1802*, and *LSEI_2570*), meaning a *ldcA*-dependent regulation (*ldcA* regulation influences the regulation of other genes), and (ii) those that are differently expressed in the M*cwaR* mutant (*LSEI_0223*, *LSEI_0251*, and *LSEI_0435*), meaning a *ldcA*-independent regulation (Figure 6A). LSEI_0393 and LSEI_1909 belong to the genes possibly regulated by the TCS but, since their expression profiles varied between exponential and stationary phases, they cannot be classified in these subgroups. The 10 other genes whose transcription changed were regulated in a CwaRS TCS-independent manner (Figure 6A), meaning the involvement of additional regulatory system(s) in response to the lack of LdcA. Also, among all the tested genes, 23 were transcribed at similar levels whatever the condition (Supplementary Figure S1 and Supplementary Table S2).

### Impact of *cwaR*, *cwaS*, and *ldcA* Gene Inactivation on the Surface Proteins

Surface protein isolations by LiCl extraction were carried out for ATCC 334, M*cwaR*, M*cwaS*, and M*ldcA* mutants and analyzed by SDS-PAGE (Figure 6B). In accordance with genome annotation, no S-layer proteins were found. In the three mutant profiles, two bands, corresponding to proteins of about 40 kDa, were five-fold lower than in the ATCC 334 strain (Figure 6B). Protein sequence analysis of these spots revealed that they corresponded to LSEI_0020 and LSEI_2029 proteins, both annotated as surface antigens. This decrease was consistent with the downregulation of LSEI_0020 and LSEI_2029 genes in mutants (see above). The protein encoded by LSEI_0020 is orthologous to Msp2 (also named p40) of *L. rhamnosus* GG (Yan et al., 2013) and *L. casei* BL23 (85 and 95% identities, respectively). The LSEI_2029 protein shares 72% identity with LGG_02016, a surface antigen (NLP/P60) of *L. rhamnosus*. The N-terminal part of LSEI_2029 protein sequence presents 46% identity with the 224 first amino acids of LSEI_0020, whereas the C-terminal part presents 50% identity with the 112 amino acids of the C-terminal part of LSEI_0281, the Msp1 (p75) ortholog. The other differences in protein profiles between ATCC 334 and mutants correspond mainly to intracellular proteins which could be more easily released from mutants because of an increased susceptibility to the extraction procedure.

### Impact of Gene Inactivation on Some Deleterious Factors Characterizing the Gut

Adhesion to eukaryotic components is a bacterial strategy to escape gut transit and interact with the gut. Thus, *L. paracasei* ATCC 334 and M*cwaR*, M*cwaS*, and M*ldcA* mutants were tested for their ability to adhere to polystyrene coated with mucin. Adhesion to mucin was similar for the ATCC 334 and mutant strains (Supplementary Figure S2). Thus, the change in PG composition and the decrease of surface antigens did not impact the mucin adhesion ability of *L. paracasei*.

**FIGURE 7:**
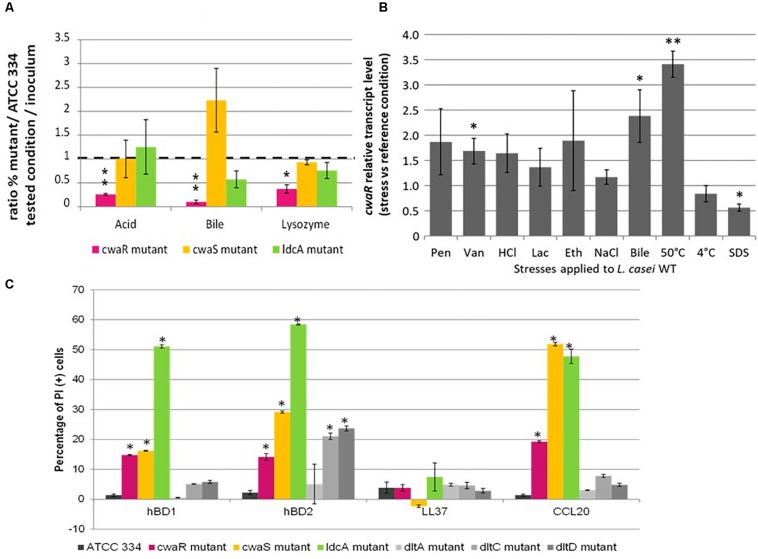
Role of *cwaSR* genes in adaptation to gut conditions and to reference stresses. **(A)** Competition in stressful conditions between *L. paracasei* mutants (M*cwaR*, M*cwaS*, and M*ldcA*) and ATCC 334. The competitions were carried out in diluted MRS broth (1/20) in 16 h at 37°C; acid pH 3.5; bile 3 g.l^–1^; lysozyme 10 g.l^–1^. The percentage was determined by counting on Petri dishes. Three biological repeats were performed, and bars indicate standard deviations. Statistical analysis was performed using the unpaired Student *t-*test: ^∗^*P* < 0.01; ^∗∗^*P* < 0.001. **(B)** Relative transcript levels of *cwaR* gene in *L. paracasei* ATCC 3334 under stressful conditions. *L. paracasei* was grown until OD reached 0.6. Then, a 15-min stress was applied. Transcript levels are expressed as the relative fold change, with *L. paracasei* cells incubated at 37°C as the reference condition (fold change = 1). Pen, penicillin 0.1 μg/ml; Van, vancomycin 0.5 mg/ml; HCl, HCl added to reach pH 3; Lac, lactic acid added to reach pH 3; Eth, ethanol 15% (vol/vol); NaCl, NaCl 1 M; Bile, bile 3 g/l; SDS, SDS 0.5 g/l. Four biological repeats were performed, and bars indicate standard deviations; ^∗^*p* < 0.05; ^∗∗^*p* < 0.01. **(C)** Percentage of PI (+) bacteria (permeabilized cell) after treatment with hBD1, hBD2, LL37, and CCL20. Values were obtained by flow cytometry. ^∗^ Indicate significant differences (*P* < 0.05).

The resistance of the three mutants to stresses encountered in the ileum was assessed by competition with ATCC 334 in the following conditions: (i) low-nutrient, acidic (pH 3.5); (ii) low-nutrient, bile-supplemented (3 g.l^–1^); and (iii) low-nutrient, lysozyme-supplemented (10 g.l^–1^) (Figure 7A). The three mutants are not equal regarding stress resistance. The M*cwaR* mutant was less resistant than ATCC 334 for all the conditions tested, contrary to M*cwaS* and M*ldcA* mutants which did not display any significant change compared to ATCC 334. These results demonstrated that, taken separately, ileal physicochemical conditions are not enough to decrease the survival of the three mutants. They also suggest a specific involvement of the CwaRS TCS in response to stress, regardless of cell surface modifications. This proposal was confirmed by measuring the relative transcript level of the *cwaR* gene, taken as a reporter for the involvement of CwaRS during stress responses. Ten abiotic stresses were applied to ATCC 334 (penicillin, vancomycin, HCl, lactate, ethanol, NaCl, bile, heat, cold, SDS) as described in the section “Materials and Methods.” Transcript levels of *cwaR* increased when vancomycin or bile were added in the medium and also during a hot thermal stress, whereas transcript levels decreased in the presence of SDS, compared to the non-stressful reference condition (Figure 7B). Thus, the CwaRS TCS could be recruited during gut transit in response to bile.

The effect of antimicrobial peptides, hBD1, hBD2, LL37, and CCL20 on the three mutants and *L. paracasei* ATCC 334 was assessed (Figure 7C). The M*dltA*, M*dltC*, and M*dltD* mutants were added to this test as predicted susceptible mutants because LTA D-alanylation reduces the negative net charge of the cell wall surface, allowing the resistance against antimicrobial cationic peptides (Neuhaus and Baddiley, 2003). None of the mutants displayed an increased susceptibility to LL37. All mutants (except M*dltA*) were more susceptible to hBD2 than ATCC334. Moreover, the M*cwaR*, M*cwaS*, and M*ldcA* mutants were susceptible to hBD1 and CCL20. The M*ldcA* mutant was even more susceptible to hBD1 and hBD2 than the other mutants. The three mutants of the operon were at least eight-fold more susceptible to hBD1 than the ATCC 334 and at least six-fold more susceptible to hBD2 and CCL20. The antimicrobial cationic peptide susceptibility could be responsible for the decreased survival of the mutants observed in the ligated rabbit ileal loop (Licandro-Seraut et al., 2014).

## Discussion

The symbiotic balance of the host with its microbiome is based on the regulation of the microbiota by the host immune system, and inversely of the immune system by its microbiota. Actors responsible for the regulation of the microbiota are increasingly well understood (cationic peptides, cathelicidin, …). The different species that comprise the microbiota must resist these different attacks, and also the stressful conditions of the gut. The *cwaRS-ldcA* operon of *L. paracasei* ATCC 334 is required to maintain the bacterium in the gut (Licandro-Seraut et al., 2014).

PG composition analysis suggests that LdcA enzyme acts as an L,D-carboxypeptidase, and not as a D-Ala-D-Ala carboxypeptidase. The same difference between predictive and empiric functions was observed for DacB of *Lactococcus lactis* (Courtin et al., 2006). Also, the inactivation of *ldcA* did not alter cell morphology, contrary to what was observed for other PG hydrolases such as LSEI_0281 (Licandro-Seraut et al., 2014), a gene whose orthologs, p75 and *msp1*, have been studied in *L. casei* BL23 and in *L. rhamnosus* GG, respectively (Claes et al., 2012; Regulski et al., 2012). Moreover, according to our observations, PG thickness was unchanged in the M*ldcA* mutant.

The *cwaRS* genes (encoding the TCS) are in an operon with the *ldcA* gene, so we hypothesized that they were related to cell wall biogenesis regulation. This assumption was confirmed by transcriptomic analysis. The CwaRS TCS is able to regulate the expression of 17 cell wall-related genes including genes encoding enzymes dedicated to lipid II synthesis. On the other hand, CwaRS TCS-independent genes encode mainly enzymes dedicated to PG assembly. This is probably the consequence of PG architecture modifications due to the inactivation of the LdcA enzyme. In addition to its cell wall-related function, the CwaRS TCS is required to resist several stresses since the M*cwaR* mutant was less able to survive in bile, acid, or lysozyme than ATCC 334 or the M*ldcA* mutant. This particular phenotype is consistent with previous reports on *L. casei* (Alcantara et al., 2011). The best candidate for the regulation of CwaRS TCS-independent genes is the LSEI_2808-2807 TCS because it is structurally close to the WalKR TCS, a TCS playing a role in cell wall metabolism in other bacterial genera (Bisicchia et al., 2007; Dubrac et al., 2007; Delaune et al., 2011).

A global downregulation of predicted PG hydrolases was observed in the M*ldcA* mutant, particularly during the stationary phase of growth. It can be assumed that this mutant required less PG hydrolases because of its PG structure (absence of relevant muropeptide substrate) or because of a lower PG turnover. The genes *LSEI_0020*, *LSEI_2029*, and *LSEI_2226* encode PG hydrolases with no predicted specificity. LSEI_2226 N-terminal domain is homologous to the *L. rhamnosus* putative cell wall hydrolase LGG_02225. These genes presented the strongest downregulations in the M*ldcA* mutant. As they are similarly downregulated in the TCS mutants (M*cwaR* and M*cwaS* mutants), which are more susceptible to stress than the M*ldcA* mutant, their lower transcriptions cannot be correlated with resistance to stress. Nevertheless, a differential posttranscriptional regulation between mutants cannot be excluded. Indeed, PG hydrolase activity can be modulated by their own *O*-glycosylation (Lebeer et al., 2012; Rolain et al., 2013) or by PG modifications such as NAG *O*-acetylation (Bernard et al., 2011), NAG *N*-acetylation (Meyrand et al., 2007), or by teichoic acid D-alanylation (Steen et al., 2005; Palumbo et al., 2006).

We assumed that the change in PG structure would also affect the barrier function of the cell wall. Above all, for the mutants, the two surface antigens (LSEI_0020 and LSEI_2029) are less abundant at the surface. This can be explained by a decrease of gene expression and/or by an alteration of the anchoring at the PG layer. The first proposal is validated for both genes. The protein encoded by *LSEI_0020* is the ortholog of *p40* (or *msp2*) that can bind mucin (Bauerl et al., 2010). Nevertheless, in our conditions, no statistically significant differences between mutants and ATCC 334 were observed for adhesion to mucin. In *L. rhamnosus*, several intestinal epithelium homeostasis-promoting functions were attributed to p40. The p40 protein can activate Akt [protein kinase B (PKB)], inhibit cytokine-induced epithelial cell apoptosis, induce colon epithelial cell growth, and decrease epithelial barrier damage induced by hydrogen peroxide (Yan et al., 2007; Seth et al., 2008). The protein encoded by *LSEI_2029* is the ortholog of the *L. rhamnosus* LGG_02016 antigen (NlpC/P60 family) which is overexpressed in an acidic environment (Koponen et al., 2012). The mutant for *LSEI_2029* is not altered in gut establishment in the rabbit ligated ileal loop model (Licandro-Seraut et al., 2014), suggesting that the decrease of LSEI_2029 antigen presentation alone is not enough to explain TCS and M*ldcA* mutant phenotypes in the rabbit gut. It is interesting to note that in *L. casei* ATCC 393, there is no LSEI_2029 homolog, and the LBCZ_0019 (the LSEI_0020 homolog) expression is three times lower and predicted to be secreted (Johnson et al., 2016). The phenotype of the three mutants can be explained by either the change in PG composition or the decreased presentation of the two antigens, or the combination of these two changes to the *L. paracasei* cell surface architecture.

The M*ldcA* mutant did not display an increased susceptibility to gut stresses currently tested, but the three *cwaRS-ldcA* mutants are more susceptible than ATCC 334 to hBD1, hBD2, and CCL20 antimicrobial peptides. Thus, the lack of LdcA modifies PG structure without changing the global surface charge, which increases susceptibility to AMPs. This phenomenon is independent of the functionality of CwaRS TCS. This could entirely explain the three mutant deficiencies in gut establishment. It makes sense regarding the mechanism of action of AMPs in Gram-positive bacteria. AMPs pass through the PG and inhibit PG synthesis. Indeed, PG precursors are activated and transported through the plasma membrane. The presence of AMP in the plasma membrane can disturb the translocation of these precursors and the PG organization (transglycosylation and transpeptidation) (Yeaman and Yount, 2003). *L. paracasei* ATCC 334 is resistant to hBD2, whereas *L. rhamnosus* GG and *L. delbrueckii* subsp. *bulgaricus* are susceptible (De Keersmaecker et al., 2006; Hugo et al., 2012). However, some other cell envelope changes could occur simultaneously, such as cell membrane structural changes in addition to PG alteration (which could also the sensitivity to nisin and AMPs). Indeed, PG structural perturbation may well be the result in a generalized cell envelope stress response which would be governed by other sensor/response systems in addition to this operon system and have a more global impact on gene expression. In this case, cell envelope stress response could be caused by structural damage.

Our results reinforce the importance of cell wall envelope in the host–microbe interaction and in bacterial adaptation to environmental changes since it is the foundation for an optimal cell wall structure (including cell wall-anchored proteins) (Sengupta et al., 2013; Delhaye et al., 2019). Deficiency in gut establishment for other *L. paracasei* mutants we have previously identified (Licandro-Seraut et al., 2014) could also result from PG modification: (i) the mutants for genes related to biogenesis of the cell wall (*dlt* operon, the *LSEI_0238* and *LSEI_2546* polysaccharide transporters); (ii) the *ldhL1 (LSEI_2549)* mutant which should be impacted in the production of lactate, a PG constituent (Ferain et al., 1996; Rico et al., 2008); (iii) the *ansB* (Asn synthase) mutant found in our screening which is reported to present an altered PG and to lose immune-activating capacity (Ito et al., 2014).

## Conclusion

The *cwaRS-ldcA* operon is composed of a functional TCS involved in the regulation of PG synthesis and a PBP which acts as a putative L,D-carboxypeptidase instead of a D-Ala-D-Ala carboxypeptidase as predicted. This operon seems to be necessary for antigen presentation and for a suitable architecture of the bacterial surface. Hence, thanks to the CwaRS TCS and LdcA, *L. paracasei* cells are able to model their surface architecture in a manner that allows them to establish in the gut and to resist host defenses.

## Data Availability Statement

All datasets generated for this study are included in the article/Supplementary Material.

## Author Contributions

HS, TP, IB, J-FC, PS, and HL contributed to study conception and design. HS, TP, AP, RW, and CP performed the experimentation and acquisition of data. HS and HL did the analysis and interpretation of data. HS drafted of manuscript. HL carried out critical revision. All authors read and approved the final manuscript.

## Conflict of Interest

The authors declare that the research was conducted in the absence of any commercial or financial relationships that could be construed as a potential conflict of interest.
