# Supplementary material for: Study of the cwaRS-ldcA Operon Coding a Two-Component System and a Putative L,D-Carboxypeptidase in Lactobacillus paracasei
Source: Front Microbiol. 2020 Mar 3;11:156. doi: 10.3389/fmicb.2020.00156 (PMC7062640; doi:10.3389/fmicb.2020.00156)
Supplement: Supplementary file 1 [file Data_Sheet_1.pdf]

Supplementary Material

Table S1: qRT-PCR primers

Name	Sequence (5' -> 3')
LCLEUSF	GCTGGATGCTGGTATTGCTT
LCLEUSR	ATCACGCAGTTTGCCTTCAT
LCRECGF	CGTAGCGTGATTCTGGTGAC
LCRECGR	TTCCAAAGATTGCTGCTTCA
LSEI_0143F	ATCACCAAATCGCCGTAGTC
LSEI_0143R	GTCATTGAAGCCAACAACCTG
LSEI_0219F	CGGTAAGGAAGCCATTACTAA
LSEI_0219R	CAACATCCATCCCAGACATATTA
LSEI_0220F	TCGCAGTGAAGAAGAGCTACTC
LSEI_0220R	TGGTCGATTTAACCTTGGTGTA
LSEI_0221F	CAGGTGGCACAACATTTACTG
LSEI_0221R	CGCATCACTGGTTCCCTTAC
LSEI_0223F	CGGCTGCTATTATTGGTGCT
LSEI_0223R	CGGCAACTTTCGTATGCTCC
LSEI_0233F	GACTGGTGGATTGCTTTGGT
LSEI_0233R	GGCTACCCGTCACATTACC
LSEI_0234F2	CTGGAAGTGGGCAGCAGTTA
LSEI_0234R2	TGTTAGCGGAATCTGGCGAA
LSEI_0236F	CTCAAAGAAGCACGGACGAG
LSEI_0236R	ATGACGACTGAATGCCAAGC
LSEI_0249F	CCGTAACCCAAACCAGACC
LSEI_0249R	CGACAGAAACAACCACCAAC
LSEI_0251F	CCAAAGGCGATTTAGTTGCTT

LSEI_0251R	CAATGAGATCCCAGCCACG
LSEI_0281F	GCCTGGTGATTTGCTCTTCT
LSEI_0281R	CTGGTTGTGGTGCCTGAATC
LSEI_0314F	GCCGTCAGTGTCTTGCTTT
LSEI_0314R	GGAACCGCCCACAATCTC
LSEI_0393F	GTAATGGCAGCAGATGTGGA
LSEI_0393R	GCTTTGACCGTTTGGAGAAA
LSEI_0435F	GACGCCTCTATGTCGAGCTT
LSEI_0435R	GCCGTTTACCCTTCAAACAA
LSEI_0539F	AATTTGGAGCAGATGCCAGT
LSEI_0539R	CTTCGCTTCGGTAATGATGG
LSEI_0708F	GGTCCTGTTCATTCATCTGCT
LSEI_0708R	TCGCTTACTCAAGGCTGTCA
LSEI_0799F2	GGAAATGACCGCTGAAGAAA
LSEI_0799R2	CATCCGTTTGCCTCCAAC
LSEI_0810F	AAGTGCGGCTTGTATTGGAA
LSEI_0810R	CGGTTTATTCGGCGGCTAT
LSEI_0906F	GCGACGAGGAAAGAAACAAT
LSEI_0906R	AAATCGGCAAATACCTGTGG
LSEI_1003F	CGCCACCTTTACGCTCAAA
LSEI_1003R	AAGACCGACCCGCAAGAAG
LSEI_1072F	CCAAGTGTTGCTGATGGGTA
LSEI_1072R	CTGCTCGTTGTCGGCTTCT
LSEI_1089F	GGCGATCCATCAACTAGGC
LSEI_1089R	TAACACAAATAGACTCAGGGC
LSEI_1110F	CCTTACCAAACGAGCGAGAA
LSEI_1110R	TTCAAGCGACGATACCCTTC
LSEI_1116F	GTATGACAACGACAGTGAATCG
LSEI_1116R	TCCTTAGCGGCACGAACT
LSEI_1152F3	TTCCCACACACAACCACTTT

LSEI_1152R3	GTCATCAACGCCTGTTTCAG
LSEI_1153F	GCGACTGTAAGGCGTAAACC
LSEI_1153R	TGGATTTGGGACGGCAAT
LSEI_1269F	CGTGACTCGCTTGTTGAAGA
LSEI_1269R	GGGTGATACTTGCCACTTTGA
LSEI_1270F	TGTTGGCAGTGGTTGCTATC
LSEI_1270R	CTGGCTGTTTCGGTGACATAA
LSEI_1271F	GATTATCACTGAACCCGAGTT
LSEI_1271R	ATCAAGCCAATCAGCGTGGT
LSEI_1272F	TGCCCAAAGTCTAACGAAACA
LSEI_1272R	TCTTCCATCAAGGTCACAACC
LSEI_1314F	ACTTTCGGCTAAGGCAGATG
LSEI_1314R	CCACGACCAATGTAATCAAGA
LSEI_1351F	AAACGCATCCAGTCAATCGTG
LSEI_1351R	CAGAGTCGCCAATAGCAGTG
LSEI_1481F	GTGATGCCGAACTCCAACA
LSEI_1481R	GGTCCAAACCGAAATGACTC
LSEI_1536F	TGACCAACCTTCGCAATG
LSEI_1536R	TGACCGCATACCAATCATCT
LSEI_1554F	GGCAAGAACCAGTCGGTATT
LSEI_1554R	CGCAGCATCTCCTGTTATGA
LSEI_1662F	ACCTGCGTTTGACTCTTGGA
LSEI_1662R	TGGCTATGCTCGGAATGAC
LSEI_1711F	CGTGGAAGTAAGCATCAAAGC
LSEI_1711R	TTATGGGATTAGTGACCGTGA
LSEI_1725F	GCTTCTTCGTTGTTAGCGTCA
LSEI_1725R	CGGGTTCTCTGCTTGGTTTA
LSEI_1802F	CTGTGTGCTTCAGGTTGAGC
LSEI_1802R	CGTGTTACTTGCTGGTGTGA
LSEI_1805F	CGTCAGAAAGCGAAGCACTC
LSEI_1805R	GGACAGGCAAGCAAGTTACC
LSEI_1909F	GTAGGTCTTGCCGCTGTCA

LSEI_1909R	TGCTGGTGGACTTGATGGTA
LSEI_2029F	CGGTGCTGGTTGAAGAAGTAG
LSEI_2029R	AAAGATAAGTTGGCTCAGTTGG
LSEI_2137F	AAGTTATGGGTGTGCGTCAG
LSEI_2137R	GGGATTGTGCGTCAAGTTAC
LSEI_2226F	TAGCAGCAACGCCACTGTAG
LSEI_2226R	ACGACACAACCAACCCAAAG
LSEI_2550F2	CTGGATGCCGATTGATTACA
LSEI_2550R2	GTCTGGTGGGTTGTTGACG
LSEI_2553F	GTTGCCAGTTTGTGTTGTCG
LSEI_2553R	TTTGTGGTTGATAGAGCGATAG
LSEI_2560F	GCACGATAGGTCACACCGTA
LSEI_2560R	GATTCCAACCACACCGTTTC
LSEI_2563F2	CAATCCGTGACTCGCTTTC
LSEI_2563R2	GCACTATTATCCGCTTGATGA
LSEI_2570F	GTGTTCATTGCTGACGGAAG
LSEI_2570R	TAGGGCGGTTTGGTAAAGC
LSEI_2677F	CTAAGCGGGTTTGAATCGTAT
LSEI_2677R	GATGATGAGTCTGGGCGTCA
LSEI_2773F	TGGGCATCAGGGACATCT
LSEI_2773R	CATAGGCTCGTTGGTCAGC

Table S2. Tested genes in transcriptomic analysis and corresponding annotation in *L. paracasei* ATCC 334 genome.

gene	function
LSEI_0020	surface antigen
LSEI_0143	D-alanine-D-alanine ligase and related ATP-grasp enzymes
LSEI_0223	UDP-N-acetylmuramyl tripeptide synthase
LSEI_0233	Glycosyltransferases involved in cell wall biogenesis
LSEI_0234	Glycosyltransferases involved in cell wall biogenesis
LSEI_0236	Lyzozyme M1 (1,4-beta-N-acetylmuramidase)
LSEI_0249	Cell wall-associated hydrolase
LSEI_0251	Glycosyltransferases involved in cell wall biogenesis
LSEI_0281	Cell wall-associated hydrolases (invasion-associated proteins)
LSEI_0314	Cell division protein FtsI/penicillin-binding protein 2
LSEI_0393	Lyzozyme M1 (1,4-beta-N-acetylmuramidase)
LSEI_0435	Beta-lactamase class C and other penicillin binding proteins
LSEI_0539	Cell wall-associated hydrolases (invasion-associated proteins)
LSEI_0599	1,4-beta-N-acetylmuramidase
LSEI_0708	Glycosyltransferases involved in cell wall biogenesis
LSEI_0799	Xanthosine triphosphate pyrophosphatase
LSEI_0810	Membrane carboxypeptidase (penicillin-binding protein)
LSEI_0906	Uncharacterized bacitracin resistance protein
LSEI_1003	UDP-N-acetylmuramate dehydrogenase
LSEI_1072	Lyzozyme M1 (1,4-beta-N-acetylmuramidase)
LSEI_1089	Glycosyltransferases involved in cell wall biogenesis
LSEI_1110	Glycosyltransferases involved in cell wall biogenesis
LSEI_1116	Muramidase (flagellum-specific)
LSEI_1152	Beta-lactamase class C and other penicillin binding proteins
LSEI_1153	UDP-N-acetylmuramyl tripeptide synthase
LSEI_1269	Cell division protein FtsI/penicillin-binding protein 2

LSEI_1270	UDP-N-acetylmuramyl pentapeptide phosphotransferase/UDP-N-acetylglucosamine-1-phosphate transferase
LSEI_1271	UDP-N-acetylmuramoylalanine-D-glutamate ligase
LSEI_1272	UDP-N-acetylglucosamine:LPS N-acetylglucosamine transferase
LSEI_1314	Bacterial cell division membrane protein
LSEI_1351	Predicted acyltransferases
LSEI_1481	Membrane carboxypeptidase (penicillin-binding protein)
LSEI_1536	N-acetylmuramoyl-L-alanine amidase
LSEI_1554	N-formylglutamate amidohydrolase
LSEI_1662	Cell division protein FtsI/penicillin-binding protein 2
LSEI_1711	UDP-N-acetylmuramate-alanine ligase
LSEI_1725	Membrane carboxypeptidase (penicillin-binding protein)
LSEI_1802	Beta-lactamase class C and other penicillin binding proteins
LSEI_1805	Beta-lactamase class C and other penicillin binding proteins
LSEI_1909	1,4-beta-N-acetylmuramidase
LSEI_2029	Cell wall-associated hydrolases (invasion-associated proteins), surface antigen
LSEI_2137	Beta-lactamase class C and other penicillin binding proteins
LSEI_2226	Cell wall-associated hydrolase
LSEI_2550	Uncharacterized protein conserved in bacteria
LSEI_2553	hypothetical protein
LSEI_2560	Alanine racemase
LSEI_2563	UDP-N-acetylmuramyl pentapeptide synthase
LSEI_2570	UDP-N-acetylglucosamine enolpyruvyl transferase
LSEI_2677	Glycosyltransferases involved in cell wall biogenesis
LSEI_2773	Beta-lactamase class C and other penicillin binding proteins

Table S3 GenBank number of the species used for comparison of the organization in different Lactobacillus species.

***Lactobacillus* species**	**GenBank number**
Lactobacillus casei	NC_010999.1
Lactobacillus paracasei	NC_008526.1
Lactobacillus buchneri	NC_018610.1
Lactobacillus brevis	NC_008497.1
Lactobacillus fermentum	NC_010610.1
Lactobacillus plantarum	NC_004567.2
Lactobacillus rhamnosus	NC_013198.1
Lactobacillus ruminis	NC_015975.1
Lactobacillus amylovorus	NC_015214.1
Lactobacillus amylolyticus	NZ_CP020457.1
Lactobacillus acidophilus	NC_006814.3
Lactobacillus crispatus	NC_014106.1
Lactobacillus delbrueckii	NC_008054.1
Lactobacillus gasseri	NC_008530.1
Lactobacillus helveticus	NZ_CP012383.1
Lactobacillus iners	NZ_GG700801.1
Lactobacillus jensenii	NZ_CP018809.1
Lactobacillus johnsonii	AE017198.1
Lactobacillus reuteri	NC_009513.1
Lactobacillus salivarius	NC_007929.1
Lactobacillus vaginalis	GCF_000159435.1

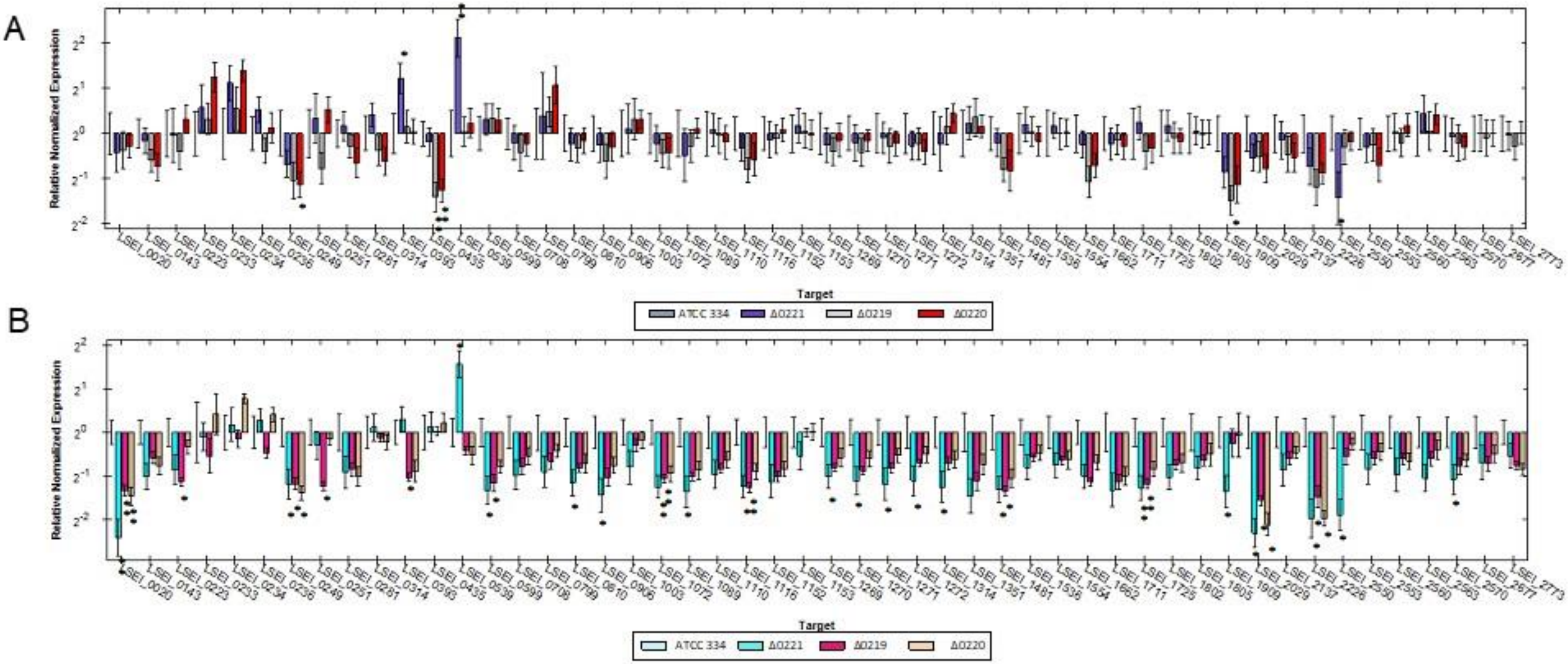

Figure S1. Relative transcript levels of genes implicated in peptidoglycan synthesis in M***cwaR*** **,** M***cwaS*** **,** M***ldcA*** **mutants compared to the *L. paracasei* parental strain ATCC 334 grown either until exponential (A) or stationary (B) phases.** Values are the mean RTL obtained for three biological repeats. Statistical analysis was performed using the unpaired Student *t* test; **, p<0.01; *, p<0.05.

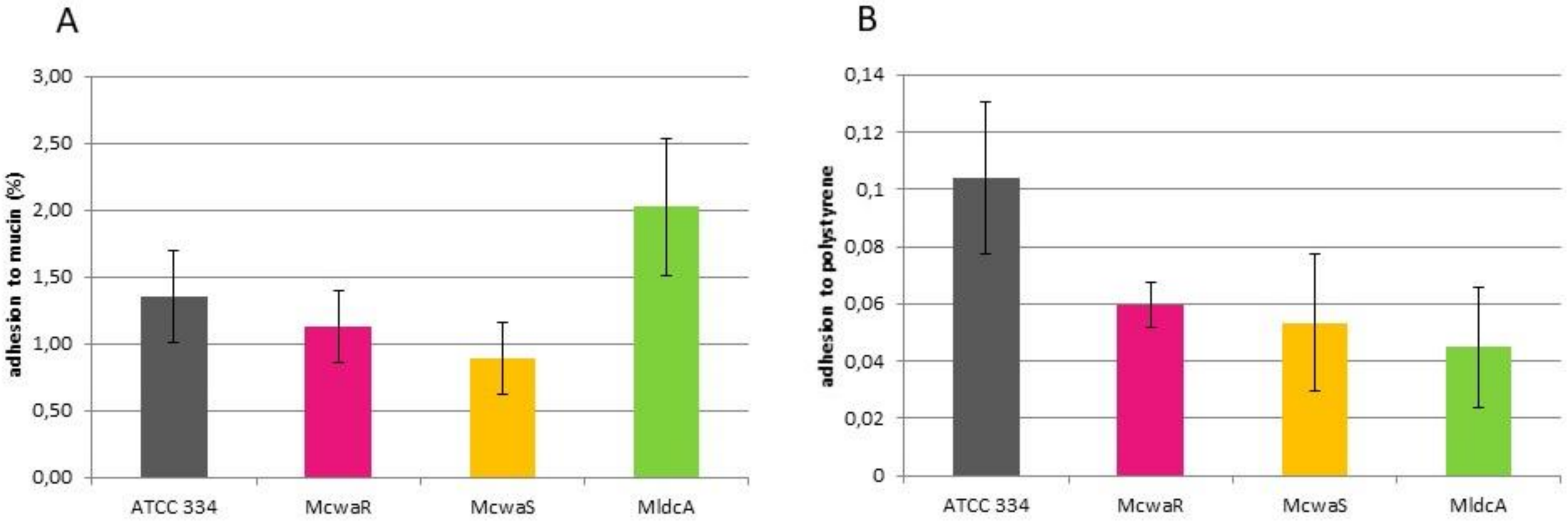

Figure S2. Adhesion of M*cwaR*, M*cwaS*, M*ldcA* mutants and ATCC 334 to polystyrene coated or not with mucin. Adhesion percentage was determined after an incubation of 3.5 h for polystyrene coated with mucin (A) and 48 h for uncoated polystyrene (B).
